# A Case of Obstinate Regurgitation of the Contents of the Stomach, Treated by the Inhalation of Chloroform; with Remarks on Regurgitation

**Published:** 1856-12

**Authors:** Isaac E. Taylor

**Affiliations:** Physician to Bellevue Hospital, New York


### SELECTIONS.

*A Case of Obstinate Regurgitation of the Contents of the Stomach, Treated by the Inhalation of Chloroform; with Remarks on Regurgitation.* By ISAAC E. TAYLOR, M.D. Physician to Bellevue Hospital, New York. Read before the New York Medical Association, and published by request.

September 1, 1855, I was requested to visit a married lady, æt. twenty-seven years, who had suffered from chronic vomiting for several weeks.

She had just returned from Newport, R.I. where she had gone to enjoy the benefit of sea-bathing for the more complete restoration of her health, which had been delicate for the last few years, but had somewhat improved the few months previous to her going to Newport. Whilst she was at Newport, the affection of the stomach commenced. In the early part of August (I think the 6th) she had been bathing, and walked from the beach to her residence, a distance of one mile, which fatigued her very much. This, with the unpleasant information she had received by letter after her return, from a near relative, and producing some excitement of mind, was believed to be the exciting cause of the malady.

When I visited her, I found her looking cheerful, and her mental energies lively as usual. No depression of spirits; there was not much, if any, emaciation since I last saw her (July 19;) there was more feebleness; pulse natural; skin the same in feel and function; tongue clean and looked natural. The countenance did not present the appearance of any serious disease of the stomach or of the abdominal organs. Appetite was good, and she enjoyed her food with some relish. The bowels were not regular, nor had they been for some time, which was the reverse of what formerly had been the case, having been subjected to a form of chronic diarrhœa for the last two or three years, at intervals. She had been married for several years; was of a leuco-phlegmatic temperament, and had had five or six miscarriages. A sub-acute ovaritis had existed, and which, under treatment, yielded during the spring. The diarrhœa also ceased previous to her leaving for Newport. She vomited, or rather regurgitated her food after every meal, and whenever any fluid of the smallest quantity was taken, and seldom without something was swallowed. There was a slight burning sensation in the region of the cardiac extremity of the stomach, under the scrobiculis cordis, after she had rejected what she had taken. There was no pain on pressure over the region of the stomach; no tenderness; no enlargement was perceptible over the abdomen. No tenderness of the spine; but tenderness, the size of a quarter of a dollar, in the right iliac region, corresponding to the lumbo-abdominal nerves in this section of the abdomen. Urine natural, and no albumen. The menstrual function was normal; there was no disease of the uterus. This organ was normal in size, position, and appearance. The ovaries also the same. The food or drink was returned, with very little change, as soon as swallowed, without nausea, and without convulsive effort or change of countenance. She had not thrown off any blood. Acidity sometimes was present, and more so during the first week than ever afterwards during the time I was in attendance. The contents of the stomach, provided the patient had eaten or drank a larger quantity than usual, would be regurgitated, as a general rule, mouthful by mouthful, till the whole was eliminated. Sometimes, however, it would be rejected in larger quantities at a time. It usually occupied her from a half hour to one hour and a half to reject her food and drink. Solid food was partly digested, and other times not. She had been treated Homœopathically while at Newport, and also by the regular method of treatment.

From the appearance of the patient, and the manner in which the contents of the stomach were rejected, I considered it a case of regurgitation, and not vomiting, and that the malady appeared to be seated in the eighth pair of nerves, and the solar or semi-lunar plexus of nerves of the stomach was incidentally involved; that the stomach was not diseased in any of its tissues at the time. I did not consider it, therefore, as merely hysterical or as sympathetic of any uterine trouble, or of any internal organ. This opinion was expressed to the family the day following. The treatment, with this view of the case, was principally by those remedies that would have an influence over that portion of the nervous system. The alkaloids were first resorted to, and then the various sedative preparations. Cannabis indica, in the one-sixth of a grain, was of some service. McMunn's elixir of opii, in five-drop doses, with five drops of vinegar, repeated every few hours, after meals and during the time of meals. The various mild ferruginous preparations were tried; chloroform internally and with camphor; nitric acid upon the stomach; blisters alone and dressed with the alkaloids; epispastic, not only to the stomach, but to the spine; also dry cupping to the spine along the greater part of its tract; stimulating liniments of chloroform, with aconite, etc. were used. Nutritious enemas were also resorted to. In truth everything that could be suggested, and that was deemed advisable, was made trial of. Her diet was restricted, and cream and milk, in tea-spoonful proportions, were administered every half hour. This treatment seemed to prostrate her so much that she was permitted to eat whatever she pleased, and in this manner, although the food was regurgitated, she was sustained better, and, as I believe, only in this way. Out-door exercise was resorted to as much and as often as her strength would admit of.

As nothing I had suggested, in the treatment of the case, appeared to afford any benefit, Prof. Joseph M. Smith was invited by me to visit her, six weeks after I first saw her; and two or three weeks after this visit of Prof. Smith, Dr. C. D. Smith was invited to join Prof. Smith and myself. Under the treatment then advised, the malady still continued without any change for the better.

As no favorable result had ensued by March, she was advised to make trial of a sea voyage. She bore the sailing remarkably well, so much so as to merit the appellation of being "the best sailor aboard of the ship," and improved as respects her general strength; but she was not benefited in the least, while at sea, respecting the regurgitation, nor when she arrived at Paris. She there came under the supervision of Sir Joseph F. Olliffe, who had associated with him Professor Trousseau, during the whole of the time she resided there, which was three months. She was also visited by Prof. Rayer. The opinion of Prof. Rayer was, that it was a severe case of gastralgia. The views entertained by Sir Joseph and Prof. Trousseau were —I quote from Sir Joseph's letter to me, June 18, 1856:—"It is a case of obstinate regurgitation, unaccompanied with organic lesion; but, from its antecedents—the anterior uterine affection, the frequent miscarriages, and the manner of invasion of the malady—we both were impressed with the idea that the uterus was the *point de depart* of all the mischief. We determined, accordingly, to establish a point of irritation on that organ, by applying the actual cautery, and selected the anterior labium of the cervix, on which there existed a small ulceration." The treatment of these gentlemen proved of no avail, and the usual internal methods of treatment for affections of the stomach were also instituted with the like result. Hydropathy was tried, as well as electro-magnetism.

On the 8th July, the patient once more presented herself to my notice, without any change for the better. She was more emaciated and weighed about eighty-three pounds, as was afterwards ascertained. For several days after she returned, nothing was suggested till she had recruited from the effects of her sea voyage; and, on the 17th July, after a slight breakfast consisting of toast and tea, and as soon as she had swallowed it, and before it was regurgitated, she was brought under the partial influence of chloroform; and, as she did not reject her food after fifteen minutes, the inhalation was continued for one and a quarter hours, and during that time no regurgitation occurred. There was, while under the effects of the chloroform, considerable uneasiness and distress in the stomach, which was perceptible from the moaning of the patient and the vermicular motions of the stomach, while the food was undergoing the process of digestion. Fifteen to twenty minutes after the chloroform had been given, she regurgitated part of her food, and continued to do so from three quarters to one hour, but not to the same extent as previously. After the trial of chloroform that day, she did not appear much feebler than before it was taken. Her food, July 17, was of a light nature and smaller in quantity than she had usually partaken of: it was rejected, but not as soon or as much. The following day, July 18, she was allowed to remain quiet with the same light nourishment as the day before. On Saturday, July 19, at 2½ P.M. directly after she had eaten her dinner of beefsteak, tomatoes, potatoes, bread, and some brandy and water, the chloroform was administered, and continued till 4 P.M. one hour and a half, and to the same extent of partial anæsthesia as before. No regurgitation occurred till 4½ P.M. and then it was at long intervals and in mouthfuls, and nothing like the quantity she had eaten. The distress, judging from the moaning of the patient, and the writhing motions of the body, was more severe, and the attempt to regurgitate more evident. This, I presume, was owing to the larger quantity of food she had eaten, and of a more solid nature than before. In the disgorging of the food there was some acidity. No food that evening was allowed till next morning, July 20, when the light food she had taken for her breakfast was not cast off as soon or as much; and, during the afternoon, after another light meal for dinner, the same thing recurred but too a partial extent. At 8 P.M. she was much enfeebled, but this was attributed to her not having partaken of the usual quantity of food she had been accustomed to, and some brandy and water was advised in small doses. At 10 P.M. there was a little regurgitation, with some acidity. Ord. mixture composed of spts. ammonia comp., bi-carb. potass., nit. potass., and hydrocyanic acid in solution, and three table-spoonfuls of this mixture were given. The medicine in this quantity was retained. In one hour afterwards, as there was some nausea, it was given again and retained—a symptom in the treatment of the case which was very gratifying to perceive, for so large a quantity had not been retained as long before since she was taken sick; and although I had, during the evening, my suspicions awakened to the possibility of her improvement, I felt at this time stronger encouragement to believe my patient was in a fair way of recovery. One hour after the last dose, as my patient had not slept during the day, and but little the evening previous, I gave her a pill composed of Indian hemp ¼gr. and watery ext. of opium one gr. and one hour after this a second. After a short interval of time, as she appeared inclined to sleep, I left her till 7½ A.M. directing that some chicken-iced-jelly should be carefully and nicely prepared, which was given in tea-spoonful quantities every half hour for three hours, to be again resumed in three hours; this quantity in such a given time was continued till the next day, and then it was given in dessert-spoonful quantities with tea-spoonful doses of brandy and water. The third day, July 23, the nourishment was increased to table-spoonful proportions, every ten or fifteen minutes, till a sufficient quantity of food was taken, and with a gradual increase of other kinds of food; and under the general advice and directions given, she improved so much as to be able to leave for Lebanon Springs, August 6, and continued to improve during a sojourn of four weeks in the mountain region, so as to gain fifteen pounds during that time; and she is at this time enjoying as good health as previously; nor has any further evidence of the malady manifested itself from the time it ceased, July 20, to the present moment.

### REMARKS.

The case of regurgitation of the contents of the stomach, as related, and which is considered as a peculiar morbid affection of that viscus, suggests a few remarks on the subject of regurgitation. In the various treatises on the practice of medicine, this form of affection of the stomach is not alluded to. Affections of this nature are usually embraced and considered as cases of vomiting, the stomach having the appearance of, and therefore believed to be, the principal seat of the disease. I think, however, it will be ascertained, on more mature reflection, and the investigation of cases of this character, that the disease is differently located. It is a conceded point, that there may be different antiperistaltic actions to which the alimentary canal, from the pharynx to the small intestines, is subjected. Rumination claims the first notice. Regurgitation is next presented to our observation, then vomiting, and, if carried further down the intestinal tract, ileus. The first two will more especially invite our attention.

Rumination, we are aware, is generally allied to the animal kingdom as well as regurgitation, and it might seem singular that the natural process of some of the animal creation might or could occur in the human species, and be recognized as one of disease. Nevertheless, such appears to be the fact, as instances of this nature do occasionally present themselves to the experience of professional men, not only in affections of the stomach, but in other parts of the human organization—the nervous system especially. By rumination, we understand the act or process by which a part of the food, after it is taken, rises into the pharynx, after a longer or shorter interval of time, varying from a few minutes to several hours.

Fluids are more easily thrown up than solids, and vegetable than animal matter. There is no nausea nor offensive taste perceptible, and after the substances have been retained for a time in the mouth, they are again swallowed, when another portion rises; and this is repeated more or less frequently in a period of time varying from a few minutes to one or two hours. The act, however, is not generally the actual rumination of the cloven-footed animals.

In the instance of a young gentleman, who has lately presented himself to my observation, this function has existed for nearly a year, but it is only in the morning after breakfast, and not during any other meal. Portions of the food he has partaken of will be returned to the mouth and then swallowed. It is sometimes thrown off or rather regurgitated. His health, in other respects, appears to be generally good. He is engaged as a clerk in business, and frequently, through the hurry or press of business, eats with rapidity, or, in other words, bolts his food.

Sir Henry Marsh, in an article on regurgitation, in the *Dublin Quarterly Journal of Medical Science*, Vol. XXIII. refers to the case of a young gentleman which bears pointedly on this affection. The young gentleman was a clerk in a bank, enjoyed good health, lived at his desk, took but little exercise, and dined hurriedly, scarcely allowing himself time to masticate his food. Soon after dinner, portions of food, with little or no effort on his part, ascended into his mouth, were re-masticated and again swallowed. In this manner, according to his own account, the whole food underwent the secondary process. It was a source of much enjoyment to him, and he prided himself upon the possession of this novel capability. Romberg, however, says he has not found the rumination in man to be actually the same as in animals, and that the morsel thrown up is not subjected again to trituration. This corresponds with my own experience. The case of Sir Henry is, nevertheless, very clear and decided in this respect, showing that the normal act of the animal kingdom may, through disease in the human species, assume the same features. In man, the act of rumination is generally an involuntary one. In a case, mentioned by Blumenback, and one also by Peter Frank, it was considered as dependent on the will. It is related by Dr. Heiling, that Professor Westendorf, of Gustrow, was able to ruminate whenever he wished, but that if he took any active exercise after meals, it would certainly occur. It may also be remarked, that if another disease should supervene, such for instance as gout or rheumatism, the affection might cease. Pressure with both hands on the stomach, we know, if freely made after a meal, will produce the same effect.

Peter Frank was of the opinion, that in persons who suffer from eructations, and are voracious, rumination is induced by a bad habit, the ruminating matter offering an encouragement to the repetition of the experiment. By regurgitation is meant that act or process by which the contents of the stomach are eliminated, whether of the fluid, gaseous, or solid nature, without much or any nausea, without any or very little convulsive effort, without sickness, or any change of color or tinge appearing in the face during the act; in truth, having the appearance as though the fluid or solid contents of the stomach issued from the stomach without any difficulty or exertion. In the same individual, the food may be sometimes vomited and sometimes regurgitated; and it may be cast off entirely, and sometimes discharged, as in the case of our patient, mouthful by mouthful till the whole is rejected, occupying from half an hour to one and a half or two hours. The food is therefore in some instances partly digested from the length of time it is being regurgitated; and will sometimes not be digested, and the act of regurgitation may then be difficult to recognize from vomiting.

Regurgitation, as well as rumination, are normal and natural functions of various forms of the animal creation. In many of the lower order of the polypi, the entire order of the entozoa, the sterelmintha of Rudolphi, the superfluous part of the food is regurgitated after the nutritious portion is abstracted by digestion. In fishes, the same is likewise noticed. In birds it is also recognized, such, for instance, as the vulture, eagle, the pheasant, parrot, and the pigeon, and it is strikingly manifest, as we have all seen, in the ruminating class, as the ox, cow, sheep, etc. The act of regurgitation differs in different animals, and which is owing to the physical structure of the muscular fibres of the œsophagus, whether they are of the striated or non-striated form. In the rodentia, according to Edward Weber, he has invariably found the muscular fibres of organic life which present both longitudinal and transverse stria, while in birds the muscular fibres are non-striated and organic: in the former, the movement is entirely that of animal muscles; in the latter, of organic muscles. In cats and dogs they are of a mixed character, the movements accordingly correspond with the double type. Judging from these investigations, it resolves itself into the proposition, that those muscles that contract and relax are of the striated form, and those that contract slowly and in a vermicular manner present the non-striated form. It is but lately we have learnt the movements of the œsophagus in health in man, and the important part it plays in vomiting or disease of the stomach.

According to the observations of Muller, and Majendie, and Budge, rhythmical contractions of the lower portions of the œsophagus take place independent of the act of swallowing, lasting about thirty seconds, and continuing longer the fuller the stomach may be. The contraction passes gradually into relaxation, which is again followed by contraction, so that the cardiac orifice of the œsophagus is not always closed equally and perfectly. Though experiments have shown that when the œsophagus is in perfect order no food can pass into it; and, in the act of vomiting, the experiments of Legallois, Beclard, and Majendie have, during the act of digestion, demonstrated the antiperistaltic motion of the œsophagus the reverse of that which it excites in deglutition. In the numerous experiments of Dr. Reid, it frequently happened that muscular contractions of the œsophagus, induced by irritating the par vagum in the neck, extended over the cardiac extremity of the stomach, but they were slower, more prolonged, and vermicular, than in the œsophagus. In the pheasant and pigeon there is, however, a change that takes place in the internal surface of the crop, at the time the young are hatched, and at this time the pigeon regurgitates the milky secretion of the crop to nourish her young with. In the rumantia, while the first two stomachs have the power of returning the food to the mouth for rumination, the third stomach, by which the act of vomiting is performed, is with great difficulty excited to exert its movement. Regurgitation is also a natural act in some instances in man, as is frequently noticed in the nursing infant, when its stomach is repleted; a portion of the milk is regurgitated, and the process of digestion then goes on to completion; and in the adult we notice in some cases, where large quantities of gases are generated, and which, on being thrown up, leave the individual easy, that digestion is then accomplished afterwards with more comfort. We recognize the salutary influence, also, in the rejection of morbid fluids which mark certain forms of indigestion, as in pyrosis. In pertussis, which is considered by many pathologists as an affection of the par vagum, engaging those portions that go to the chest and œsophagus. In this disease it is noticed that, after the cough, a part and sometimes the whole contents of the stomach are regurgitated by the child, and as soon as rejected, the child will again resume its eating, immediately after the paroxysm; and it is rejected without much or any effort, nausea, or convulsive action of the stomach.

From the intractable nature of the cases we are discoursing upon, continuing, as they may, for several years, provided no organic lesion should spring up—and we can record one instance of two, and another of three years duration, which recovered without any lesion; in cases of this nature, the mind of the medical man will naturally grow incredulous respecting the true character of the disease, and will again and again feel strongly inclined to suppose or believe that organic changes have occurred, or must exist, where such a lengthened continuance of a disease has shown itself, and that the various forms of degeneration of that organ, either of the fibrous and cancerous order, and perforating ulcer, or ulceration, may be present, and even sarcinæ. If this view is not entertained, and various forms of treatment been adopted and proved of little or no avail, hysteria generally comes in for a solution of the difficulty, as throwing more light on the subject, for want of more satisfactory explanation, presuming there is "something behind" not yet clearly recognized, being either idiopathic or sympathetic of some uterine or ovarian trouble, or possibly from some internal organ —such as the liver, kidney, or tuberculous affection of the brain, or a general tuberculosis. Emesis, in diseases of this nature, occurs not only during the time of digestion, but between the time of eating, and possibly some slight hemorrhage may be manifest, as adding to the solution of the disease; this, however, is not the case in instances of regurgitation; for in nearly every instance, in which regurgitation occurs, the food is immediately, or very soon after it is partaken of, rejected, and whilst it is undergoing the process of digestion. Believing that this one symptom of diagnosis will be sufficient, together with the history of the case, while the food is rejected, and the slight emaciation that occurs, to draw attention to that peculiar morbid condition of the stomach, I will not discuss the further diagnostic symptoms incident to the other morbid affections of that organ.

Entertaining the opinion, from the first time the patient came under my charge, that the symptoms were those of regurgitation and not of vomiting; that the affection was seated in the par vagum, and more especially its cardiac extremity, and the stomach was only partially implicated, and that through the ganglionic or solar plexus of nerves, and that it was believed to be free from any disorganization of its tissues. The indications, therefore, of treatment would be directed to overcome the irritution of that portion of the nervous system to obtain a favorable result. At the instance of Sir Joseph F. Olliffe, during the month of May last, this opinion was communicated to himself and Professor Trousseau, under whose care the patient was during her sojourn in Paris for three months. Having taken this view of the nature of the affection which my patient was affected with, let us see what reason there was why such an opinion should be entertained, and for the kind of treatment which was adopted, and which has been the means or instrument of the restoration of the invalid to her general state of health.

We are aware that the par vagi arises from the corpora olivare and restiform, that it sends many fibres over a large space and to many organs, such as the external ear, pharynx, larynx, œsophagus, trachea, heart, and lungs, and some to the stomach, and also to other viscera of the abdomen. In their course they communicate with the sympathetic, and to the cerebro-spinal nerves to a partial extent. It is considered by Stilling, Vankempen, Muller, Reid, and others, as a motor and sensiferous nerve; and that it has been proved by Dr. Reid, and other physiologists previous to him, that irritation of the trunk of the vagus excited motions in the œsophagus which extended over the cardiac portion of the stomach; and as Muller has remarked, that the motions of the œsophagus are dependent on the motor fibres of the vagus, and are probably excited by impressions made on the sensitive fibres of that nerve. The vagi has no motor influence on the nerve of the stomach, for neither by galvanic influence nor by mechanical irritation applied to the neck, can motions of the stomach be excited; but lesion of the vagi is generally followed by rejection of food, loathing of it, and arrestment of the digestive process, as has been incontrovertibly shown by numerous experimenters.

Digestion may, however, occur, though the nerve has been divided in the neck, even when the extremities are an inch apart; and that when divided, and in contact, the digestion was more perfect; and that if irritated by pinching, etc. digestion was more easily accomplished. We are, therefore, justified in concluding that a deleterious influence is propagated downwards to the stomach by lesion of the vagi, either by pressure on it, or by disease.

In the tenth and eleventh experiments of Dr. Reid, on the vagi of dogs, the vomiting was very frequent, and the animal would vomit up its food, afterwards partake of it again, and then reject it again, and this continued for several minutes. The death of the animal was attributed to the frequency of vomiting; and, on examination after death, there was evidence that digestion was proceeding at the time of dissolution. As the experiments are of so much interest, and bear so clearly and pointedly on the subject, as illustrating the function of that nerve while under lesion, I have thought it might not be amiss to quote the eleventh experiment, as exhibiting the close analogy to cases of regurgitation in man.

The subject of the experiment was an old and large pointer. The vagi was cut on the 27th June, at half-past eight A.M. On the 28th, he was dull and languid; had no apparent uneasiness; refused to eat, and had a little vomiting. On the 29th, took a little food, which was soon vomited. On the 30th, was dull, and vomited a small quantity of food taken. 2d July, took a quantity of milk, which was vomited some time afterwards. 3d, appetite deficient, and the food taken was vomited after a shorter or longer time. On the 5th, still vomited a great part of the food taken, and was evidently becoming more feeble. At 3 P.M. was poisoned with prussic acid, and died soon afterwards. On post mortem examination, soon after death, the stomach was found healthy and slightly red on the inner surfaces, as is usual when digestion is proceeding. This was also the case in two other experiments of a like nature. After lesion of the vagi, vomiting, or rather regurgitation, is generally occasioned by the presence of the food in the stomach, and not from any irritation of the nerve itself, even if it is divided; and it has been remarked that dogs made no effort to vomit when the stomach was empty, but were seized with vomiting immediately or soon after food was taken into the stomach, where lesion of the vagi existed. It is very apparent, therefore, from the experiments so carefully carried on by the eminent physiologists referred to, that there is a marked and distinct difference between the physiological action of the nerves going to the stomach and the œsophagus; and that when vomiting is produced by the lesion of any internal organ, it is by a reflex action, transmitted from the irritation of the brain to the medulla oblongta first, and then reflected to the stomach; but that cases of vomiting, or rather of regurgitation, may exist idiopathically when the lesion of the par vagi is solely affected, as we have shown; and further, of the central organs, the spinal cord rarely bears an etiological relation to vomiting, for injuries and diseases of that portion of the nervous system are so uniformly followed by paralysis of the expiratory muscles, that that alone accounts, according to Romberg, for the rare occurrence of vomiting as a concomitant symptom. In the observations by Olliver, there are but few instances, and they apply rather to the first stage of myelitis, than to other spinal affections; but, on the other hand, the brain bears a close relation to vomiting, for we have often seen how a mental impression quickly suffices to produce it. Instances of voluntary vomiting are very rare, though it is recorded that Bichat possessed this faculty.

When I reflected on the views that have been promulgated of the natural action of the œsophagus, the result of the experiments on the par vagum in animals, where there was a lesion of that nerve; seeing the persistency of the regurgitation of my patient, with the slight emaciation when I first saw her, and noticing also the ease with which she rejected her food, of what ever form it might consist, of fluid or solid, and occurring at the time the food was taken, and seldom or ever without something was partaken of, though of the slightest or smallest quantity, it was evident that the case presented the appearance so nearly allied to the par vagum, where lesion had occurred from experiments on that nerve in animals, and that that nerve had therefore undergone a lesion in some form or other. Being unsuccessful in overcoming or mitigating the affection whilst she was under my charge, by treatment, previous to her sailing for Europe, and then returning without having received any benefit from the treatment of several of the most prominent professional gentlemen in Paris, with the views I entertained, I deemed it advisable that chloroform should be tried after the patient had partaken of food, and the reasons for this method of treatment I will now relate:—

The par vagum was considered as the *point de depart* of the malady, with the solar plexus of nerves going to the stomach incidentally engaged, and that in consequence of this the *antiperistaltic motion* of the *œsophagus* had *occurred;* that the *stomach* had *ceased* to *perform* its *natural* functions perfectly, and that it was possible, if the *antiperistaltic* action of the *œsophagus* could be *arrested* or *quieted* after *food* had been taken, thus permitting the stomach to assume its natural and perfect functions, which it had not done for nearly a year, and the digestion process sustained for one or one and a half hours without regurgitation, more nourishment would be taken into the system than she had received during the time she was usually engaged in disgorging her food as she was accustomed to do: for this must have been the only way in which she had existed, or else she would have died of inanition. The first trial we have noticed, July 17, was in some measure successful and warranted a repetition of the remedy; and, on the 19th July, after a full meal for her dinner, proved to be so successful that there was no occasion to resort to it again, nor to any other remedial agent, except a few doses of the mixture as stated above.

I am loath to believe, with the views I have expressed, why there was functional lesion of the eighth pair of nerves, and of that portion going to the cardiac extremity of the stomach, corroborated by the experiments of so many eminent physiologists, they could be viewed as speculative in their character, and the treatment considered as of the same order, for this view must, I think, be negatived by the success which has attended the administration of the remedy, while the stomach was repleted, proving, I think, the correctness of the reasoning and as excluding the malady from that protean form of disease, hysteria, under which so many affections are classed, for want of a better and more scientific explanation.

It might be alleged, that as the mind was materially affected at its commencement, and as one of the exciting causes, the mental impression may have sustained the affection, but this can have no validity, as the circumstances that existed at the commencement and previous to its occurrence, still continue as at that time.

It might be supposed that the sea voyage on her return home had conduced to the favorable result of the case, but this could not reasonably be alleged as aiding in the least to the benefit of the patient, for the affection continued in the same manner as previous to her leaving America; nor did the quiet and rest which she enjoyed at home aid her, as no improvement had manifested itself till the inhalation of the chloroform had been resorted to, two weeks afterwards.

I am not unmindful, however, that changes may occur in the cardiac extremity of the stomach, when the disease has existed for a considerable length of time, and that the œsophagus may, in its tissue, take on the degenerative form. In three ruminating individuals, all of the male sex, Arnold found the œsophagus in the stomach considerably dilated, limited above by a constriction, so as to form a genuine cardiac vestibule—the muscular layers of the stomach and œsophagus were strongly developed, and in two of the cases they were larger and wider than usual. In the first instance the internal branch of the accessory of Willis was much larger than usual, so as almost to equal the *external branch,* a relation similar to what is found in ruminating animals. Joseph Frank relates a case of a Professor, at Wilna, who died, after suffering some few years from vomiting, and, on examination, the vagi were found surrounded on each side by a steatomatous tumor. In a female, three months advanced in utero-gestation, who was subjected to much vomiting, thirty or forty times a-day, reported by Lobstein, the semi-lunar ganglion with the par vagum was in a high state of irritation. The stomach was found perfectly healthy on post mortem examination. There is, therefore, no question that the sources of reflex irritation, proceeding from the sensory fibres of the vagi, occupy a prominent rank.

In conclusion, I need not say I have experienced no ordinary pleasure in presenting this interesting case to the attention of the Association, with the few remarks on regurgitation, and the reasons for the pathology of the malady, and why this method of treatment was adopted. I think it exhibits the application of a remedial agent through physiological deduction, and not through a mere empirical manner, or as a mere hysterical affection, and serves to prove the great benefit we have derived from the patient and scientific results of the experiments of so many eminent physiologists, and as the presentation of a new method of treatment in affections of this nature, and I should apprehend of others of a like character, as for instance in those of utero-gestation which sometimes assume the same phase.—*New York Jour. of Med.*

